# Hepatitis C Virus in Vietnam: High Prevalence of Infection in Dialysis and Multi-Transfused Patients Involving Diverse and Novel Virus Variants

**DOI:** 10.1371/journal.pone.0041266

**Published:** 2012-08-14

**Authors:** Linda Dunford, Michael J. Carr, Jonathan Dean, Allison Waters, Linh Thuy Nguyen, Thu Hong Ta Thi, Lan Anh Bui Thi, Huy Duong Do, Thu Thuy Duong Thi, Ha Thu Nguyen, Trinh Thi Diem Do, Quynh Phuong Luu, Jeff Connell, Suzie Coughlan, Hien Tran Nguyen, William W. Hall, Lan Anh Nguyen Thi

**Affiliations:** 1 Ireland Vietnam Blood-Borne Virus Initiative (IVVI), Dublin, Ireland and Ha Noi Vietnam; 2 National Virus Reference Laboratory, University College Dublin, Dublin, Ireland; 3 Laboratory for Molecular Diagnostics, National Institute of Hygiene and Epidemiology, Ha Noi, Vietnam; University of Cincinnati College of Medicine, United States of America

## Abstract

Hepatitis C virus (HCV) is a genetically diverse pathogen infecting approximately 2–3% of the world's population. Herein, we describe results of a large, multicentre serological and molecular epidemiological study cataloguing the prevalence and genetic diversity of HCV in five regions of Vietnam; Ha Noi, Hai Phong, Da Nang, Khanh Hoa and Can Tho. Individuals (n = 8654) with varying risk factors for infection were analysed for the presence of HCV Ab/Ag and, in a subset of positive specimens, for HCV RNA levels (n = 475) and genotype (n = 282). In lower risk individuals, including voluntary blood donors, military recruits and pregnant women, the prevalence of infection was 0.5% (n = 26/5250). Prevalence rates were significantly higher (*p*<0.001) in intravenous drug users (IDUs; 55.6%, n = 556/1000), dialysis patients (26.6%, n = 153/575) commercial sex workers (CSWs; 8.7%, n = 87/1000), and recipients of multiple blood transfusions (6.0%, n = 32/529). The prevalence of HCV in dialysis patients varied but remained high in all regions (11–43%) and was associated with the receipt of blood transfusions [OR: 2.08 (1.85–2.34), *p* = 0.001], time from first transfusion [OR: 1.07 (1.01–1.13), *p* = 0.023], duration of dialysis [OR: 1.31 (1.19–1.43), *p*<0.001] and male gender [OR: 1.60 (1.06–2.41), *p* = 0.026]. Phylogenetic analysis revealed high genetic diversity, particularly amongst dialysis and multi-transfused patients, identifying subtypes 1a (33%), 1b (27%), 2a (0.4%), 3a (0.7%), 3b (1.1%), 6a (18.8%), 6e (6.0%), 6h (4.6%), 6l (6.4%) and 2 clusters of novel genotype 6 variants (2.1%). HCV genotype 1 predominated in Vietnam (60%, n = 169/282) but the proportion of infections attributable to genotype 1 varied between regions and risk groups and, in the Southern part of Vietnam, genotype 6 viruses dominated in dialysis and multi-transfused patients (73.9%). This study confirms a high prevalence of HCV infection in Vietnamese IDUs and, notably, reveals high levels of HCV infection associated with dialysis and blood transfusion.

## Introduction

Hepatitis C virus (HCV) has been estimated to infect approximately 2–3% of the world's population, with highest prevalence rates occurring in low and middle income regions including Africa and Southeast Asia [1]–[4]. Transmission of HCV involves direct exposure to contaminated blood and is associated with intravenous drug use, iatrogenic exposures, tattooing, body piercing and, less frequently, through vertical transmission and high risk sexual behaviour [1], [4]–[8]. Iatrogenic routes of transmission implicated in HCV infection include blood transfusions, surgical and dental procedures, dialysis, acupuncture, needlestick injury and use of unsterilised needles [4], [9]–[17]. The latter has been highlighted in Egypt where nationwide treatment for schistosomiasis under suboptimal hygiene conditions from 1960 to 1987 has resulted in national HCV seroprevalence rates of approximately 14% [12], [18].

In industrialised countries, iatrogenic transmission of HCV is now rare and the burden of HCV infection is largely restricted to intravenous drug user (IDU) populations [18]. In contrast, iatrogenic transmission of HCV still occurs frequently in many resource-limited settings as a result of inadequate screening and failure to implement universal precautions [13], [17], [18]. Dialysis has been associated with transmission of HCV and prevalence rates as high as 18% in the Asia-Pacific region have been reported [17], [19]–[21]. Transmission is more commonly associated with haemodialysis compared to peritoneal dialysis and has been shown to be increased following longer durations of dialysis and increased frequency of blood transfusion [10], [11], [17].

Chronic HCV infection is associated with high levels of morbidity and mortality and long term sequelae [22], [23]. Studies have reported that up to 80% of infections may progress to chronic infection, of which 10–20% will result in the development fibrosis and cirrhosis, and up to 5% will develop hepatocellular carcinoma (HCC) [18], [23]. Chronic HCV infection also has significant socio-economic implications, which are compounded by restrictions in access to expensive therapies and the lack of an effective vaccine. Co-morbidities associated with alcohol consumption and coinfection with other blood-borne viruses such as HBV and HIV are also associated with accelerated progression of liver disease [24]–[28].

HCV is a positive polarity, single-stranded RNA virus of the family *Flaviviridae*, genus *Hepacivirus* and is classified into six major groups (genotypes 1–6), each containing a variable number of more closely related distinct subtypes. Due to the absence of proof reading by the virally encoded RNA dependent RNA polymerase, the virus evolves rapidly and sequence diversity is extremely high, with 31–33% divergence between genotypes and 20–25% between subtypes [29]. Genotype 6 viruses are the most genetically diverse HCV genotype, with 23 subtypes currently recognised. New strains continue to be identified, with subtypes 6v and 6w recently described from China and Taiwan respectively [30], [31]. Although some genotypes are widely distributed, there are clear epidemiological and geographical patterns associated with other genotypes such as the predominance of genotype 6 in Southeast Asia and Southern China [32].

There are limited studies regarding HCV infection in Vietnam and recently a call to action for nationwide screening of hepatitis viruses was published [33]. Prevalence rates in Vietnam appear to vary between regions and risk groups, ranging from 1–2.9% in the general population [33]–[35] to 46–87% in IDUs [16], [33], [36]. A broad distribution of HCV genotypes have been reported in Vietnam, with genotypes 1 and 6 predominating [37]–[39]. In the present study we have attempted to establish a catalogue of HCV infection in Vietnam by carrying out a large serological and molecular epidemiological study in different geographic regions and involving individuals with different risk factors for infection.

## Materials and Methods

### Ethics Statement

Ethical approval for the study was obtained from the National Institute of Hygiene and Epidemiology (NIHE) in Ha Noi. All specimens and survey information were obtained with informed, written consent and subsequently anonymised.

### Study Group

This cross-sectional study serologically investigated 8654 specimens for HCV infection collected from eight different population groups including IDUs, commercial sex workers (CSWs), blood donors, military recruits, pregnant women, dialysis patients, elective surgery patients and recipients of multiple blood transfusions. Paired serum and plasma specimens were obtained during 2008 and 2009, along with detailed demographic information, from five sites in Vietnam: Ha Noi (n = 1750) and Hai Phong (n = 1750) in the North, Da Nang (n = 1750) in the Central region and Khanh Hoa (n = 1725) and Can Tho (n = 1679) in the South.

### Viral Serology

All specimens were tested using a commercially available enzyme immunoassay (EIA) for HCV using the Monolisa Ag/Ab HCV Ultra (Bio-Rad Laboratories, CA, USA).

### Quantitative RT-PCR

A representative subset of HCV Ab/Ag positive specimens (n = 475), which included all serological positives from dialysis and multi-transfused patients, were selected for molecular analysis. Nucleic acid was extracted from 140 µl of plasma from HCV Ab/Ag positive specimens (n = 475), using the QIAamp Viral RNA minikit (Qiagen, Crawley, UK), as per the manufacturer's instructions. Brome mosaic virus (BMV) RNA (5 pg/specimen) was included during the extraction as an exogenous internal control to ensure sample addition and the absence of PCR inhibitors. HCV viral load (VL) was determined using a quantitative real-time reverse transcriptase polymerase chain reaction (qRT-PCR). BMV RNA was co-amplified as an internal control and serial dilutions of a plasmid-derived HCV RNA standard were used to prepare the standard curve. The PCR reaction was performed with previously published primers targeting the HCV 5′ untranslated region (UTR) [40]. Briefly, 5 µl of extracted RNA was reverse transcribed and amplified in qRT-PCR with 0.6 µM forward primer, 0.8 µM reverse primer and 0.4 µM probe in a 25 µl total reaction volume with the Superscript III One Step RT-PCR system with Platinum Taq DNA Polymerase (Invitrogen Life Technologies, Paisley, UK) on an ABI 7500 FAST real-time platform (Applied Biosystems, Warrington, UK) with the following cycling parameters: an initial 15 min incubation at 50°C, followed by 2 min at 95°C and 45 cycles of 95°C for 15 s and 60°C for 34 s. The assay was calibrated against the WHO 3^rd^ International Reference Standard for HCV RNA (NISBC Code 06/100) and validated with a limit of detection of 300 IU/ml plasma (2.48 log_10_ IU/ml) and a linear dynamic range of 3×10^2^–8×10^7^ IU/ml (2.48–7.9 log_10_ IU/ml). The assay was optimised to ensure high concordance with a commercially available HCV Viral Load assay, giving an R^2^ value of 0.95 when correlated with the Roche COBAS-Ampliprep COBAS-Taqman HCV Viral Load Assay. Inter-assay and intra-assay coefficients of variation ranged up to a maximum of 3.5% and 6.3%, respectively.

### HCV Characterisation

For HCV genotyping, viral RNA from HCV RNA positive specimens were reverse transcribed using Superscript III RT kit and RNaseOUT (Invitrogen Life Technologies, Paisley, UK) as previously described [32]. cDNA was amplified with the Expand High Fidelity PCR system (Roche Applied Sciences, Mannheim, Germany) using 0.2 mM final concentration deoxynucleoside triphosphates (dNTPs) and 0.3 µM primers, as previously described [32], [41]. A nested PCR targeting a 377 base pair (bp) region of the HCV *NS5B* gene was performed using previously published primers [32]. First and second round amplification of the *NS5B* gene were performed with a 2 min initial denaturation at 94°C, followed by 28 cycles of 94°C for 15 s, 60°C for 30 s and 72°C for 45 s with a final extension step of 7 min at 72°C [32]. Single round amplification of a 494 bp region of the overlapping *core* and *E1* genes was performed with a 2 min initial denaturation at 94°C, followed by 40 cycles of 94°C for 15 s, 60°C for 30 s and 72°C for 45 s and a final extension step of 7 min at 72°C using previously published primers [41]. Unincorporated primers and dNTPs were removed from HCV amplicon using Exo-SAP IT (Affymetrix, Cleveland, USA) and purified products were sequenced bidirectionally on an ABI 3730 sequencing platform. Contiguous assembly of sequences was performed by Lasergene version 8 (DNASTAR, Madison, WI, USA) [42]. Genbank accession numbers are JX102664 - JX103137.

### Phylogenetic Analysis

Datasets comprising HCV *NS5B* and *core/E1* reference sequences, representing all currently assigned genotypes of HCV, were downloaded from the Los Alamos database (http://hcv.lanl.gov/content/sequence/HCV/ToolsOutline.html) and aligned with the study sequences using Bioedit version 7.05 [43]. Phylogenetic trees for both the *NS5B* and *core/E1* sequences were constructed using the neighbour joining distance method under a Kimura-2-parameter model of evolution in PAUP* version 4.0 beta10 [44]. Phylogenies were heuristically searched using a subtree pruning and regrafting perturbation algorithm. Genotypes obtained by phylogenetic analysis were confirmed using the Los Alamos online database (http://hcv.lanl.gov/content/sequence/BASIC_BLAST/basic_blast.html). To further examine sequences from the epidemic in dialysis and multi-transfused patients, a midpoint rooted phylogenetic tree of the *NS5B* region was drawn with relevant reference and patient derived sequences. A neighbour joining tree was constructed in PAUP* based on the HKY85 model of evolution, a gamma distribution and a proportion of invariable sites using the tree bisection and reconnection (TBR) rearrangement scheme in a heuristic search. To further characterise novel sequences, maximum likelihood analyses were performed for both the *NS5B* and *core/E1* gene sequences using general time reversible (GTR) submodels, a gamma distribution and a proportion of invariable sites with a representative of each subtype of genotype 6. Statistical support for all tree topologies was evaluated with 1000 bootstrap replicates. Phylogenetic trees were visualised, annotated and coloured using Figtree v1.3 (http://tree.bio.ed.ac.uk/software/figtree).

Recombination analysis of all novel sequences identified was performed with the RDP3 software using six different programs – RDP, GENECONV, MaxChi, Bootscan, Chimaera and SiScan [45]. Analysis of pairwise nucleotide similarities was performed on both the *NS5B* and *core/E1* genes of novel sequences with representative genotype 6 reference sequences (6a–6w) using Bioedit version 7.05 [43].

### Statistical Analysis

Data from the study is presented as the mean values ± standard deviation (SD) and ranges. Continuous variables, such as viral load, were compared between populations using the Student's t-test. Categorical data were analysed using the Chi-squared and odds ratio tests. Independent risk factors for HCV infection in dialysis patients were examined using logistic regression analysis and SPSS software version 18.0. *p*-values<0.05 were considered statistically significant.

## Results

### Prevalence of HCV Infection in Vietnam

**Figure 1:**
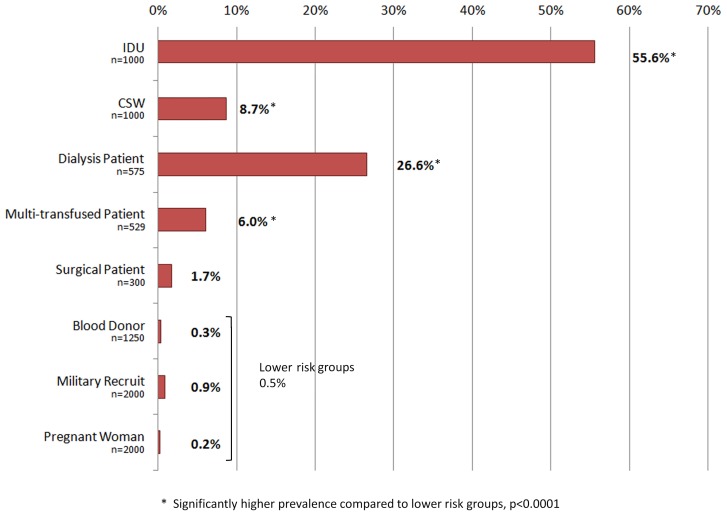
Seroepidemiology of HCV in 8 Different Vietnamese Risk Groups, n = 8654. The total percentage of HCV Ab/Ag positives in each population group is shown at the end of each bar. The number of samples tested from each group is listed below. 59.7% of CSWs testing positive for HCV also reported intravenous drug use.

In a sample of 5250 individuals considered to be at low risk for infection (voluntary blood donors, military recruits and pregnant women) the prevalence of HCV was 0.5%. Significantly higher HCV prevalence rates were identified in populations associated with higher risk activities, specifically IDUs (55.6%, n = 556/1000), dialysis patients (26.6%, n = 153/575), CSWs (8.7%, n = 87/1000) and multiply transfused patients (6.0%, n = 32/529; *p*<0.001; Figure 1). The prevalence of HCV in IDUs in Vietnam varied widely between the study sites; Ha Noi (59.5%, n = 119/200), Hai Phong (87%, n = 174/200), Da Nang (54%, n = 108/200), Khanh Hoa (20%, n = 40/200), Can Tho (57.5%, n = 115/200). Seventy-six percent (n = 166/218) of IDUs were viremic with a mean viral load of 5.25±1.05 log_10_ IU/ml (range 2.1–7.3). HCV infection in the CSW cohort may also be associated with drug use practices as 59.7% of HCV positive CSWs reported previous injecting drug use. Sixty-five percent of HCV infected CSWs (n = 40/62) were viremic and these had a mean viral load of 5.21±1.06 log_10_ IU/ml (2.78–7.6).

### Risk Factors Associated with HCV in Dialysis and Multi-transfused Patients

Univariate odds ratio (OR) calculations revealed that dialysis and multi-transfused patients were significantly more likely to have been infected with HCV than lower risk groups, [multi-transfused 12.2 (6.98–21.32), *p*<0.001; dialysis 68.7 (43.06–109.59), *p*<0.001]. The prevalence of HCV in the multi-transfused patient group (6%, n = 32/529) also varied considerably between regions: Ha Noi (13%, n = 13/100), Hai Phong (1%, n = 1/100), Da Nang (4%, n = 4/100), Khanh Hoa (5.3%, n = 8/150) and Can Tho (7.6%, n = 6/79). In dialysis patients, the prevalence rates also varied between the study sites; Ha Noi (43%), Hai Phong (11%), Da Nang (32%), Khanh Hoa (33%) and Can Tho (17.3%; Table 1). The lowest prevalence of HCV in dialysis patients was in Hai Phong (11%) where patients reported no history of blood transfusion or prior surgery and also had a shorter mean duration of dialysis (Table 1). Conversely, the highest HCV prevalence identified in a dialysis group (Ha Noi, 43%) was associated with high levels of transfusion (90.7%) and longer duration of dialysis (mean 7.3 years; Table 1).

**Table 1:** Prevalence of HCV Infection in Dialysis Patients in Vietnam, n = 575.

	Ha Noi	Hai Phong	Da Nang	Khanh Hoa	Can Tho	Total
**No. patients tested**	100	100	100	125	150	575
**HCV Ab/Ag pos**	43.0% (43)	11.0% (11)	32.0% (32)	32.8% (41)	17.3% (26)	26.6% (153)
**HCV RNA pos**	90.7% (39)	54.5% (6)	68.8% (22)	73.1% (30)	61.5% (16)	73.9% (113)
**Viral load log_10_ IU/ml**	5.0±1.0 (2.1–7.1)	3.4±1.0 (2.4–4.6)	4.2±1.2 (2.5–6.1)	4.6±1.5 (2.1–6.9)	4.7±1.2 (2.2–6.3)	4.6±1.3 (2.1–7.1)
**Variables in HCV positive patients**						
**Age, years**	49.4±13.3 (23–74)	25.2±3.5 (21–30)	44.2±12.9 (25–72)	46.4±14.7 (24–77)	49.4±15.0 (22–79)	45.7±14.6 (21–79)
**Gender, % Male**	51.2% (22)	45.5% (5)	68.8% (22)	63.4% (26)	57.7% (15)	58.8% (90)
**Years receiving dialysis**	7.3±3.1 (2–16)	1.8±0.4 (1–2)	3.9±2.0 (1–8)	2.8±1.8 (1–8)	3.3±2.4 (1–9)	4.4±3.0 (1–16)
**With history of transfusion**	90.7% (39)	0% (0)	100% (32)	82.9% (34)	69.2% (18)	80.4% (123)
**Years receiving transfusions**	7.7±4.5 (1–25)	n/a	4.2±5.2 (<1–30)	3.7±6.9 (<1–30)	3.5±2.7 (<1–9)	5.2±5.3 (<1–30)
**With history of surgery**	30.2% (13)	0% (0)	21.9% (7)	61.0% (25)	80.8% (21)	43.1% (66)

Data presented as mean ± SD (range) or % (number) as applicable.

No dialysis patients reported intravenous drug use or high risk sexual behaviour.

Pos - positive; n/a - not applicable.

**Figure 2:**
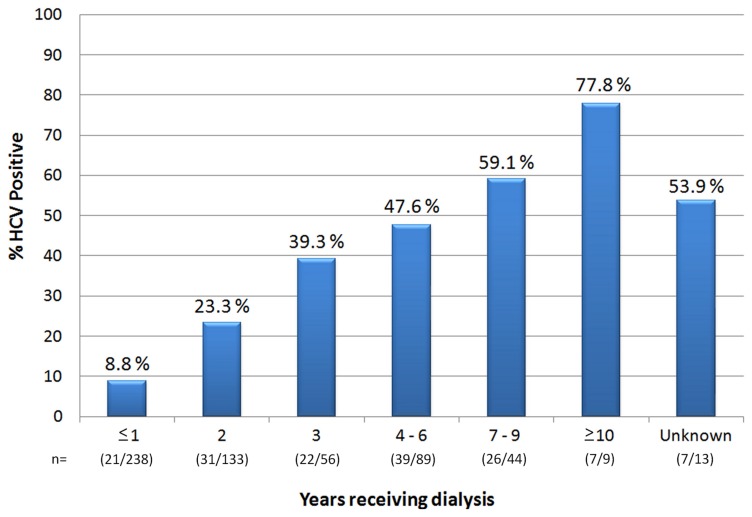
Seroepidemiology of HCV in Dialysis Patients. This figure depicts HCV prevalence in relation to duration of dialysis in years. Increased duration of dialysis is strongly associated with an increase in HCV infection (*p*<0.001).

Overall, the prevalence of HCV in non-transfused dialysis patients was 17.4% (n = 30/172), whereas, in multi-transfused dialysis patients this was significantly higher (30.5%, n = 123/403; *p*<0.001). In fact, dialysis patients receiving blood transfusions were approximately twice as likely (OR: 2.08 (0.85–5.05), *p* = 0.001) to be infected with HCV compared to non-transfused dialysis patients. A multivariate regression analysis of the risk factors associated with HCV infection in dialysis patients confirmed that the duration of dialysis was strongly associated with HCV infection (Figure 2 and Table 2). Male gender and the number of years from first blood transfusion were also significantly associated with HCV infection (Table 2).

**Table 2:** Factors Associated With HCV Infection among Dialysis Patients in Five Regions of Vietnam using Logistic Regression Analysis, n = 575.

Variable	OR	95% CI	*p* – value
**Age**	1.01	1.00–1.02	0.185
**Male Gender**	1.60	1.06–2.41	0.026*
**Duration Receiving Transfusions (per 1 year increase)**	1.07	1.01–1.13	0.023*
**Duration Receiving Dialysis (per 1 year increase)**	1.31	1.19–1.43	<0.001*
**History of Surgery**	0.98	0.64–1.50	0.936

* *p* values<0.05 deemed significant.

### HCV Genetic Diversity in Vietnam

**Figure 3:**
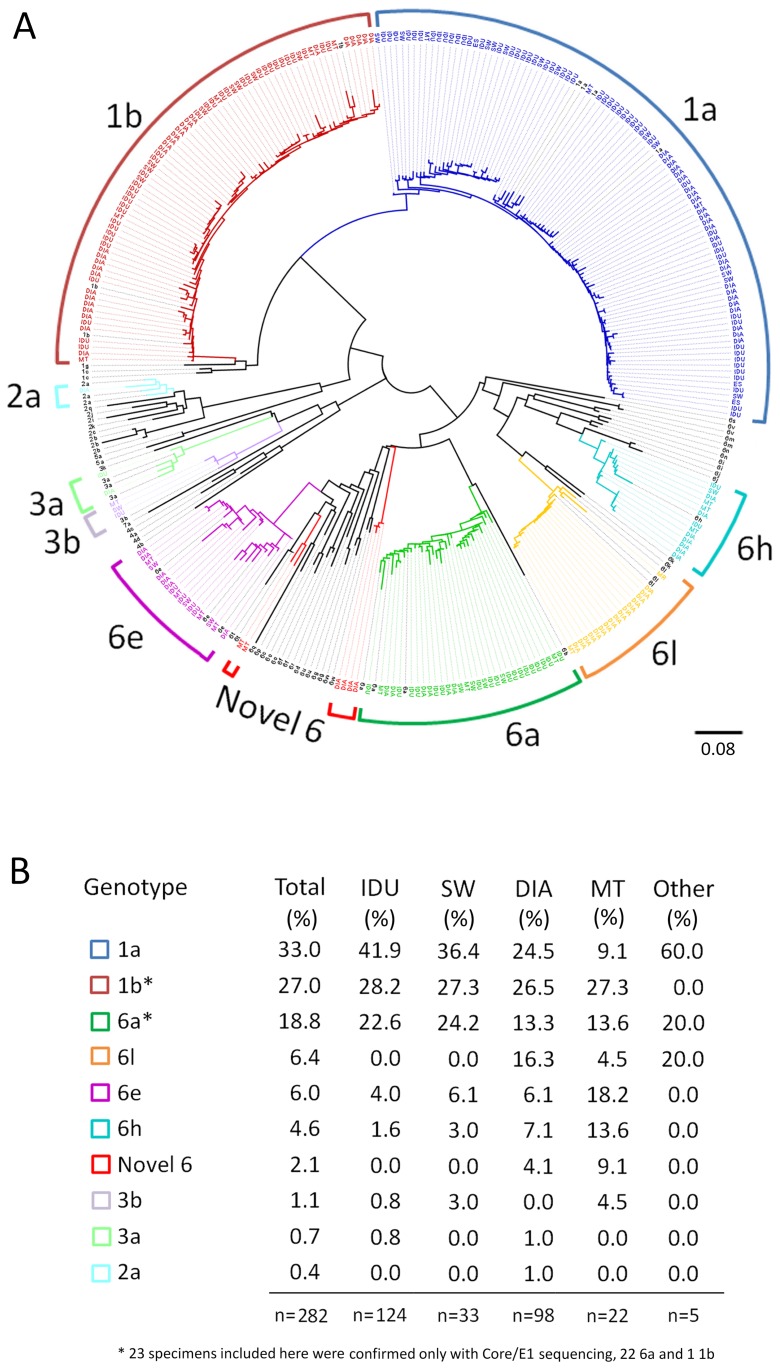
Phylogenetic Analysis of the HCV *NS5B* Gene. Vietnamese HCV gene sequences from the present study (n = 259 *NS5B*) are presented with reference sequences (n = 74) downloaded from the Los Alamos database. Analysis was based on a 329-bp of the HCV *NS5B* gene (nucleotides 8282–8610 relative to H77 NC004102). “A” depicts a midpoint rooted radial phylogenetic tree constructed using the neighbour joining distance method under a Kimura-2-paramter model of evolution. Bootstrap values >70% were obtained for all major nodes separating the confirmed genotypes (not shown). The scale bar indicates an evolutionary distance of 0.08 nucleotide substitutions per site. Branches and annotations are colour coded for all HCV subtypes identified in this study, with reference sequences shown in black. Sequences are annotated by the study cohort from which they were obtained, namely: IDU, intravenous drug user; SW, commercial sex worker; DIA, dialysis patient; MT, multi-transfused patient; ES, elective surgery patient; MR, military recruit. Reference sequences are annotated by subtype name. “B” represents all obtained HCV genotypes/subtypes in the varying risk groups. In total, genotypes were identified for 282 specimens - 201 based on both the *NS5B* and *core/E1* regions, 58 from the *NS5B* region alone and 23 from the *core/E1* region only. Genbank accession numbers are JX102664–JX103137.

Phylogenetic analysis confirmed genetically diverse HCV populations and genotype analysis for both *NS5B* and *core/E1* regions were in agreement for all specimens tested. The distribution of genotypes and subtypes by risk group are summarised in Figure 3. Genotypes 1, 6, 3 and 2 were detected in 59.9% (n = 169), 37.9% (n = 107), 1.8% (n = 5) and 0.4% (n = 1) of subjects, respectively. Genotype 1 was the most prevalent HCV genotype in our study, although this varied between regions as follows: Ha Noi (54%, n = 47/87), Hai Phong (72.1%, n = 31/43), Da Nang (81.4%, n = 35/43), Khanh Hoa (47.2, n = 25/53) and Can Tho (55.4%, n = 31/56).

Analysis of the different risk groups revealed that genotype 1 viruses accounted for a significantly higher proportion of HCV infections in both IDUs (70.1%, n = 87) and CSWs (63.7% n = 21), compared to dialysis (51%, n = 50) and multi-transfused (36.4% n = 8) patients, *p*<0.001 (Figure 3).

### Genetic Diversity of HCV in Dialysis and Multi-transfused Patients

**Figure 4:**
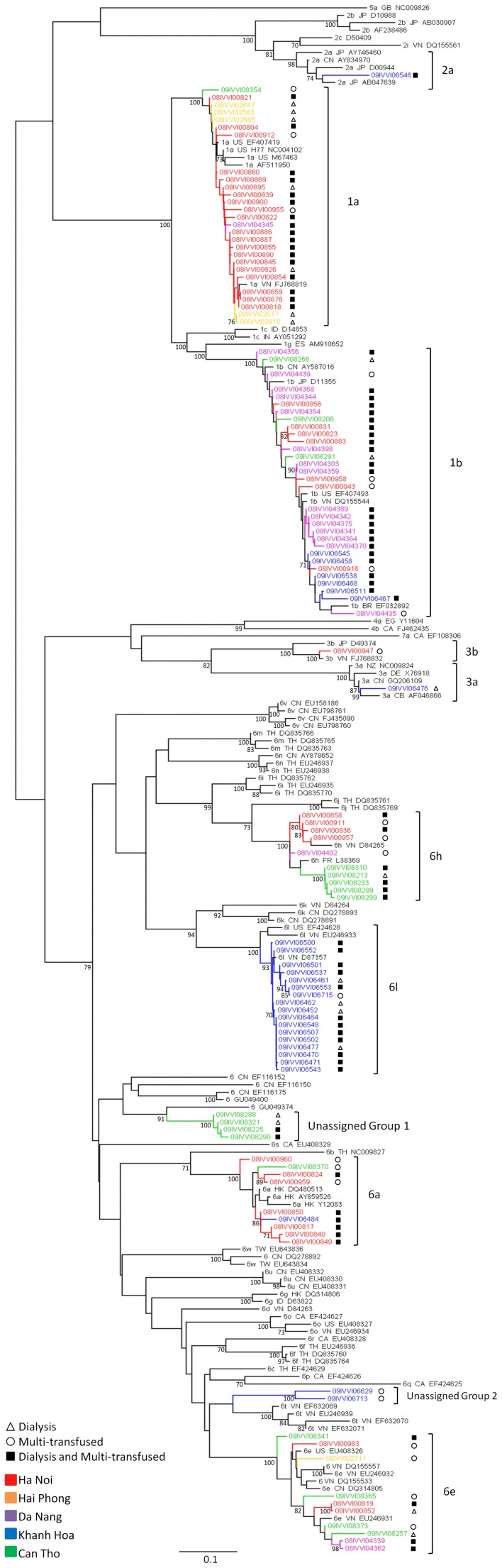
Molecular Characterisation of HCV Genotypes in Vietnamese Dialysis and Multi-transfused Patients. Analysis of HCV patient (n = 113) and reference sequences was performed on a 329-bp fragment of the HCV *NS5B* gene (nucleotides 8282–8610, numbering based on H77). A neighbour joining tree was constructed using the Kimura-2-parameter model of evolution with gamma distribution and a proportion of invariable sites. Bootstrap values over 70% are shown at the respective nodes. The scale bar indicates an evolutionary distance of 0.1 nucleotide substitutions per site. Reference sequences are annotated with their confirmed genotype and subtype, country of isolation where known and Genbank accession number. Sequences from dialysis and multi-transfused patients are represented in colour, differing by location - red for Ha Noi, orange for Hai Phong, purple for Da Nang, blue for Khanh Hoa and green for Can Tho. Open triangles are used to denote sequences from dialysis patients, open circles are used to denote sequences from multi-transfused patients and filled squares are used where a patient fits both of these criteria. Brackets select the subtypes in which sequences were identified in this study. Two unassigned groups of sequences were identified – a group of sequences obtained from four dialysis patients in Can Tho and sequences obtained from two multi transfused patients in Khanh Hoa and these are labelled as unclassified groups 1 and 2. For 6 additional specimens, 6a genotypes (5 Ha Noi and 1 Can Tho) were identified by *core/E1* sequencing.

Genotypes 1, 2, 3 and 6 were identified in dialysis and multi-transfused individuals with nine recognised subtypes (1a, 1b, 2a, 3a, 3b, 6a, 6e, 6h, 6l) and two clusters of novel sequences (Figure 4). Genotype 1 viruses were dominant in dialysis and multi-transfused patients in the Northern and Central cities of Ha Noi (64%, n = 27/42), Hai Phong (83%, n = 5/6) and Da Nang (84%, n = 16/19). In contrast, genotype 6 was the predominant virus in the Southern regions of Khanh Hoa (71%, n = 20/28) and Can Tho (78%, n = 14/18). There is clear geographical clustering of some HCV variants and high levels of sequence identity within regions. For example, there are two distinct clades of 6h viruses from Ha Noi in the North and Can Tho in the South that cluster separately with high bootstrap support (Figure 4).

A monophyletic cluster of sequences from 16 dialysis patients from Khanh Hoa was observed, branching with a 6l reference strain originally identified in Vietnam in 1994. Seventy five percent (n = 12/16) of the 6l infected dialysis patients had also received multiple transfusions and there was an additional sequence clustering with the 6l clade from a multi-transfused individual not on dialysis. The mean duration of dialysis was 2.1±1.2 years (range: 1–5) and at the time of specimen collection all patients were undergoing dialysis 2–3 times a week. Nine dialysis patients also reported a history of surgery. No other high risk factors for infection were reported and this cluster would therefore appear to be an example of HCV transmission within a dialysis unit. The specimens were all collected within a 4 week time period in 2009. In our study, subtype 6l was almost exclusively detected in Khanh Hoa and this subtype was detected in only one other individual: a military recruit from Da Nang (Figure 2). While the majority of sequences from Khanh Hoa dialysis patients were 6l (64%, n = 16/25), a number of other genotypes were also identified co-circulating in these patients including 1b, 2a, 3a and 6a, suggesting multiple introductions of HCV in dialysis centres.

### Novel Variants of HCV

**Figure 5:**
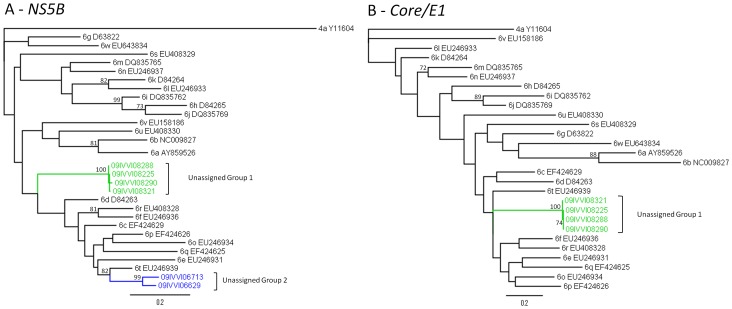
Maximum Likelihood Phylogenetic Analysis of Novel HCV Genotype 6 Sequences in both *NS5B* and *core/E1* Genes. HCV sequences identified in this study (coloured and marked by brackets) and reference sequences for all currently recognised subtypes of HCV genotype 6 are analysed with A) a 329-bp fragment of the HCV *NS5B* gene corresponding to nucleotides 8282 to 8610 of the H77 strain and B) a 446-bp fragment of the HCV *core* and *E1* genes corresponding to nucleotides 860 to 1305 of the H77 strain. Rooted trees were constructed using submodels of the general time reversible (GTR) model, a gamma distribution and a proportion of invariant sites. Bootstrap values over 70% are shown on the corresponding branches. Scale bar indicates an evolutionary distance of 0.2 substitutions per site. “Unassigned group 1” sequences were obtained from 4 dialysis patients in Can Tho and the sequences in unassigned group 2 were from multi-transfused patients in Khanh Hoa.

Novel HCV sequences forming two highly divergent clades within genotype 6 were identified with neighbour joining phylogenetic analyses (Figure 4). The two clusters of novel variants are annotated in Figures 4 and 5 as “unassigned group 1” and “unassigned group 2”. Further analysis of these sequences using maximum likelihood methods, with a representative sequence from each currently recognised subtype within HCV genotype 6 (Figure 5), confirmed that the sequences do not cluster with any other subtypes of 6 and have full bootstrap support (>99%) for clustering within their own groups. Recombination analysis of the novel variants did not demonstrate any evidence of recombination in these sequences either in the *NS5B* or *core/E1* gene regions (data not shown).

A BLAST analysis against all available HCV sequences in the Los Alamos Database found the closest similarity for “unassigned group 1” sequences was 84% to another unclassified genotype 6 strain (GU049374) in the *NS5B* region and 77% similarity in the *core/E1* region to a Vietnamese subtype 6e strain (EU246931). A pairwise comparison of nucleotide sequence identity between each of the novel sequences showed 98.7–99.6% similarity to each other in the *NS5B* region and 98.9–99.7% similarity in the *core/E1* region. When compared to the 23 currently recognised HCV genotype 6 subtypes, group 1 sequences showed nucleotide similarities of 68.0–76.5% in the *NS5B* gene and 66.8–77.2% in the *core/E1* region. The four sequences that comprised “unassigned group 1” were amplified from samples collected from male dialysis patients in Can Tho. Two patients had been receiving blood transfusions over a 5–7 year period and the other two had not received any transfusions. All four reported a history of surgery. Other than iatrogenic exposures, no other high risk activities were reported.

The two sequences in “unassigned group 2” were 81–83% similar to 6t variants (EU246939 and EU632071) in the *NS5B* gene. Unassigned group 2 sequences had only 89.9% nucleotide sequence identity to each other but consistently grouped together with 100% bootstrap support. The group 2 sequences had 65.6–83.2% similarity over the *NS5B* gene with highest nucleotide identity to 6t viruses. “Unassigned group 2” sequences were identified in two male multi-transfused patients in Khanh Hoa in 2009. Neither had undergone dialysis but one reported having a surgical procedure with no other risks reported.

## Discussion

This study confirms a high prevalence of HCV infection in Vietnamese IDUs (55.6%) and also reveals high levels of HCV infection associated with dialysis (26.6%), particularly when compounded by a history of blood transfusion. The prevalence of HCV in IDUs varied significantly between regions, ranging from 20% in Khanh Hoa to over 80% in Hai Phong. Previous estimates of HCV infection in Vietnamese IDUs have been reported to range from 19–87%, again varying by region [16], [36], [37], [46]–[48]. Indeed, a recent prospective study of IDUs in Ha Noi estimated that HCV prevalence in IDUs ranged from 30% to 70% depending on the length of time they had been injecting drugs [36].

In lower risk groups, including military recruits, voluntary blood donors and pregnant women, the prevalence of HCV was 0.5%, which is lower than in many previous reports which have ranged from 1–9% [16], [35], [46], [47]. Sexual transmission of HCV is still relatively rare and, to date, HCV transmission within CSW cohorts in Vietnam has not been well studied. Although the prevalence described in CSWs in this study (8.7%) is higher than estimates from other Asian countries, this is likely confounded by the effect of injecting drug use practices as almost 60% of the HCV positive CSW cohort reported such activity. Evidence exists to support sexual transmission of HCV, although available data suggest the efficiency of transmission by the sexual route is low [49]. Despite this, a number of studies have reported a higher seroprevalence of HCV infection in CSWs compared to the general population. Seroprevalence studies from different geographical areas have reported a range of anti-HCV levels in female sex workers: including the Democratic Republic of Congo (6.6%), Thailand (2%), Afghanistan (1.9%) and South Korea (1.4%), although, only the latter excluded intravenous drug users from their study [50]–[53].

The prevalence of HCV infection we identified in dialysis patients (26.6%, range: 11–43%) was lower than a previous Vietnamese study which identified HCV in 54% of dialysis patients in Ho Chi Minh City in 1994 [16]. However, as seen in the IDU population, the HCV prevalence in dialysis patients varied significantly from region to region, ranging from 11% in Hai Phong to 43% in Ha Noi. In some regions, namely Ha Noi, Da Nang and Khanh Hoa, the HCV prevalence in dialysis patients was higher than that reported from neighbouring Asian countries in the last decade; 5.9% in Thailand, just below 10% in Taiwan and Hong Kong, 8.5–12.5% in Japan and 18% in Shanghai, China [17], [54], [55].

Blood transfusion remains a significant risk factor for HCV infection in Vietnam and our results demonstrated that 6% of multi-transfused patients were seropositive. Moreover, the high level of infection seen in the dialysis group was compounded by a history of transfusion in 70% of patients. Indeed, the prevalence of HCV was significantly higher in those dialysis patients who had received multiple blood transfusions than in those who had not (30.5% versus 17.4%; *p*<0.001), although this certainly could also have been influenced by the increased duration of dialysis (3.1 years versus 1.9 years). In recent years, Vietnam has reported a steady rise in voluntary unpaid blood donations and blood donor screening for HIV, HBV and HCV is now mandatory [56]. The high levels of HCV infection we detected in transfused individuals could possibly have been associated with a past practice of paid blood donation and a lack of rigorous blood donation screening for transfusion transmitted infections [16], [47]. However, this remains to be established.

A significant association between increased HCV prevalence and longer duration of dialysis was established. Male gender and years from first transfusion were also seen to be independent risk factors for HCV infection in dialysis patients. These associations have been previously documented in dialysis centres in other countries [10], [11], [54]. Despite the association of HCV transmission with duration of dialysis, a high seroprevelance of 8.8% was also detected in dialysis patients that had been receiving treatment for one year or less.

Phylogenetic analysis identified high genetic diversity in circulating strains of HCV, confirming the polyphyletic nature of this virus in Vietnam. A wide variety of genotypes including 1a, 1b, 2a, 3a, 3b, 6a, 6e, 6h, 6l and novel variants were identified. Genotype 1 viruses (1a/1b) predominated throughout the country (60%) and also in the IDU (70.1%) and CSW (63.7%) cohorts. This is consistent with a recent report describing a predominance of genotype 1 in Hai Phong IDUs [37]. The high levels of genotype 1a and 1b in Vietnam are particularly significant as genotype 1-infected individuals are less likely to obtain a sustained virological response following treatment with pegylated interferon-α and ribavirin [57].

A different distribution of HCV genotypes was noted in the South of Vietnam (Khanh Hoa and Can Tho), where genotype 6 viruses were dominant in the dialysis and multi-transfused groups (73.9%). This is consistent with a recent report describing a predominance of genotype 6 in blood donors and patients with liver disease in Ho Chi Minh City [39]. Phylogenetic analysis revealed clear geographical clustering of some variants and identified a large monophyletic cluster of 6l viruses in dialysis and multi-transfused patients in Khanh Hoa. Previous reports have suggested that differences in HCV distribution between Northern and Southern regions of the country may be attributed to the fact that injecting drug use have been in widespread use for much longer in the South [58], [59]. It would also appear that the high genetic diversity observed in dialysis patients indicates multiple introductions of HCV into Vietnamese dialysis units.

New subtypes of HCV, and indeed new genotypes, continue to be identified around the world [41]. In fact, many of the new subtypes of HCV recently recognised have been first described in Vietnam [41], [60]. A proposal for a unified system of nomenclature of HCV genotypes was published in 2005 along with guidelines to define what constitutes a new subtype and these have been adopted by the three international HCV databases, Los Alamos (United States), euHCVdb (France) and the Hepatitis Virus database in Japan [29]. Provisional designation of a new subtype requires at least 3 individuals to be independently infected with viruses that differ by a minimum of 15% at the nucleotide level in both the *core/E1* and *NS5B* region, but group together consistently when analysed by a variety of phylogenetic methods [29]. Here we describe two novel variants of HCV genotype 6 in Southern Vietnam. One variant (unclassified group 1), found in 4 dialysis patients in Can Tho, has less than 77% similarity within the *NS5B* and the *core/E1* regions to previously published strains of HCV, and the other variant (unclassified group 2), found in 2 multi-transfused patients in Khanh Hoa, had 83% identity to published strains in the *NS5B* region. These new variants, in particular group 1, meet the internationally accepted criteria for designation of a new subtype on the basis of nucleotide sequence divergence; however, confirmation of this will require the identification of further cases of infection elsewhere [29]. Further studies are also required to identify the geographical spread, if any, of these potential new HCV genotype 6 variants and to ascertain their frequency in the general population.

Overall, the results of our study clearly indicate that the burden of HCV infection remains high in IDUs, dialysis patients, CSWs and multi-transfused patients in Vietnam. As no effective vaccine for HCV exists, and treatment remains prohibitively expensive, the focus of public health interventions must be on preventative initiatives such as needle exchange programmes and providing integrated, cost-effective screening to enhance disease surveillance [61]. Encouraging results from needle and syringe programmes have been recently reported in Australia where HCV prevalence rates in IDUs declined from 62% to 50% from 2008 to 2009 [62]. Primary preventative measures, together with a safe supply of blood and reinforcement of universal precautions in the health care environment should help reduce the burden of infection over time.
